# Supplementary material for: What really matters for global intergenerational mobility?
Source: PLoS One. 2024 Jun 20;19(6):e0302173. doi: 10.1371/journal.pone.0302173 (PMC11189229; doi:10.1371/journal.pone.0302173)
Supplement: S1 Appendix — (DOCX) [file pone.0302173.s001.docx]

**Appendix 1. The list of countries under this study**

| Total countries (N = 153) | Fragile | Region |
| --- | --- | --- |
| Developing economies (N=115) | | |
| Afghanistan | Yes | South Asia |
| Albania | No | Europe & Central Asia |
| Angola | No | Sub-Saharan Africa |
| Argentina | No | Latin America & Caribbean |
| Armenia | No | Europe & Central Asia |
| Azerbaijan | No | Europe & Central Asia |
| Bangladesh | No | South Asia |
| Belarus | No | Europe & Central Asia |
| Benin | No | Sub-Saharan Africa |
| Bhutan | No | South Asia |
| Bolivia | No | Latin America & Caribbean |
| Bosnia and Herzegovina | No | Europe & Central Asia |
| Botswana | No | Sub-Saharan Africa |
| Brazil | No | Latin America & Caribbean |
| Bulgaria | No | Europe & Central Asia |
| Burkina Faso | No | Sub-Saharan Africa |
| Burundi | Yes | Sub-Saharan Africa |
| Côte d'Ivoire | Yes | Sub-Saharan Africa |
| Cabo Verde | No | Sub-Saharan Africa |
| Cambodia | No | East Asia & Pacific |
| Cameroon | No | Sub-Saharan Africa |
| Central African Republic | Yes | Sub-Saharan Africa |
| Chad | Yes | Sub-Saharan Africa |
| China | No | East Asia & Pacific |
| Colombia | No | Latin America & Caribbean |
| Comoros | Yes | Sub-Saharan Africa |
| Congo, Dem. Rep. | Yes | Sub-Saharan Africa |
| Congo, Rep. | Yes | Sub-Saharan Africa |
| Costa Rica | No | Latin America & Caribbean |
| Djibouti | Yes | Middle East & North Africa |
| Dominican Republic | No | Latin America & Caribbean |
| Ecuador | No | Latin America & Caribbean |
| Egypt, Arab Rep. | No | Middle East & North Africa |
| El Salvador | No | Latin America & Caribbean |
| Eswatini | No | Sub-Saharan Africa |
| Ethiopia | No | Sub-Saharan Africa |
| Fiji | No | East Asia & Pacific |
| Gabon | No | Sub-Saharan Africa |
| Gambia | Yes | Sub-Saharan Africa |
| Georgia | No | Europe & Central Asia |
| Ghana | No | Sub-Saharan Africa |
| Guatemala | No | Latin America & Caribbean |
| Guinea | No | Sub-Saharan Africa |
| Guinea-Bissau | Yes | Sub-Saharan Africa |
| Haiti | Yes | Latin America & Caribbean |
| Honduras | No | Latin America & Caribbean |
| India | No | South Asia |
| Indonesia | No | East Asia & Pacific |
| Iran, Islamic Rep. | No | Middle East & North Africa |
| Iraq | Yes | Middle East & North Africa |
| Jordan | No | Middle East & North Africa |
| Kazakhstan | No | Europe & Central Asia |
| Kenya | No | Sub-Saharan Africa |
| Kiribati | Yes | East Asia & Pacific |
| Kosovo | Yes | Europe & Central Asia |
| Kyrgyz Republic | No | Europe & Central Asia |
| Lao PDR | No | East Asia & Pacific |
| Lebanon | Yes | Middle East & North Africa |
| Lesotho | No | Sub-Saharan Africa |
| Liberia | Yes | Sub-Saharan Africa |
| Madagascar | No | Sub-Saharan Africa |
| Malawi | No | Sub-Saharan Africa |
| Malaysia | No | East Asia & Pacific |
| Maldives | No | South Asia |
| Mali | Yes | Sub-Saharan Africa |
| Mauritania | No | Sub-Saharan Africa |
| Mauritius | No | Sub-Saharan Africa |
| Mexico | No | Latin America & Caribbean |
| Moldova | No | Europe & Central Asia |
| Mongolia | No | East Asia & Pacific |
| Montenegro | No | Europe & Central Asia |
| Morocco | No | Middle East & North Africa |
| Mozambique | Yes | Sub-Saharan Africa |
| Myanmar | Yes | East Asia & Pacific |
| Namibia | No | Sub-Saharan Africa |
| Nepal | No | South Asia |
| Nicaragua | No | Latin America & Caribbean |
| Niger | No | Sub-Saharan Africa |
| Nigeria | No | Sub-Saharan Africa |
| North Macedonia | No | Europe & Central Asia |
| Pakistan | No | South Asia |
| Papua New Guinea | Yes | East Asia & Pacific |
| Paraguay | No | Latin America & Caribbean |
| Peru | No | Latin America & Caribbean |
| Philippines | No | East Asia & Pacific |
| Romania | No | Europe & Central Asia |
| Russian Federation | No | Europe & Central Asia |
| Rwanda | No | Sub-Saharan Africa |
| São Tomé and Príncipe | No | Sub-Saharan Africa |
| Senegal | No | Sub-Saharan Africa |
| Serbia | No | Europe & Central Asia |
| Sierra Leone | No | Sub-Saharan Africa |
| Solomon Islands | Yes | East Asia & Pacific |
| South Africa | No | Sub-Saharan Africa |
| South Sudan | Yes | Sub-Saharan Africa |
| Sri Lanka | No | South Asia |
| Sudan | Yes | Sub-Saharan Africa |
| Tajikistan | No | Europe & Central Asia |
| Tanzania | No | Sub-Saharan Africa |
| Thailand | No | East Asia & Pacific |
| Timor-Leste | Yes | East Asia & Pacific |
| Togo | Yes | Sub-Saharan Africa |
| Tonga | No | East Asia & Pacific |
| Tunisia | No | Middle East & North Africa |
| Turkey | No | Europe & Central Asia |
| Tuvalu | Yes | East Asia & Pacific |
| Uganda | No | Sub-Saharan Africa |
| Ukraine | No | Europe & Central Asia |
| Uzbekistan | No | Europe & Central Asia |
| Vanuatu | No | East Asia & Pacific |
| Venezuela, RB | No | Latin America & Caribbean |
| Vietnam | No | East Asia & Pacific |
| West Bank and Gaza | Yes | Middle East & North Africa |
| Yemen, Rep. | Yes | Middle East & North Africa |
| Zambia | No | Sub-Saharan Africa |
| High-income economies (N=38) | | |
| Australia | No | East Asia & Pacific |
| Austria | No | Europe & Central Asia |
| Belgium | No | Europe & Central Asia |
| Canada | No | North America |
| Chile | No | Latin America & Caribbean |
| Croatia | No | Europe & Central Asia |
| Cyprus | No | Europe & Central Asia |
| Czech Republic | No | Europe & Central Asia |
| Denmark | No | Europe & Central Asia |
| Estonia | No | Europe & Central Asia |
| Finland | No | Europe & Central Asia |
| France | No | Europe & Central Asia |
| Germany | No | Europe & Central Asia |
| Greece | No | Europe & Central Asia |
| Hungary | No | Europe & Central Asia |
| Iceland | No | Europe & Central Asia |
| Ireland | No | Europe & Central Asia |
| Israel | No | Middle East & North Africa |
| Italy | No | Europe & Central Asia |
| Japan | No | East Asia & Pacific |
| Korea, Rep. | No | East Asia & Pacific |
| Latvia | No | Europe & Central Asia |
| Lithuania | No | Europe & Central Asia |
| Netherlands | No | Europe & Central Asia |
| New Zealand | No | East Asia & Pacific |
| Norway | No | Europe & Central Asia |
| Panama | No | Latin America & Caribbean |
| Poland | No | Europe & Central Asia |
| Portugal | No | Europe & Central Asia |
| Slovak Republic | No | Europe & Central Asia |
| Slovenia | No | Europe & Central Asia |
| Spain | No | Europe & Central Asia |
| Sweden | No | Europe & Central Asia |
| Switzerland | No | Europe & Central Asia |
| Taiwan, China | No | East Asia & Pacific |
| United Kingdom | No | Europe & Central Asia |
| United States | No | North America |
| Uruguay | No | Latin America & Caribbean |

*Source: Global Database on Intergenerational Mobility (2023), cohorts of study = 1940s, 1950s, 1960s, 1970s and 1980s*
