# Supplementary material for: What really matters for global intergenerational mobility?
Source: PLoS One. 2024 Jun 20;19(6):e0302173. doi: 10.1371/journal.pone.0302173 (PMC11189229; doi:10.1371/journal.pone.0302173)
Supplement: S2 Appendix — (DOCX) [file pone.0302173.s002.docx]

**Appendix 2. The pre-modelled dataset**

| **No** | **Variable** | **Stats / Values** | **Freqs (% of Valid)** | **Missing** |
| --- | --- | --- | --- | --- |
| 1 | mobility  [numeric] | \| Mean (sd) : 0.5 (0.2) \| \| --- \| \| min ≤ med ≤ max: \| \| 0 ≤ 0.6 ≤ 1 \| \| IQR (CV) : 0.3 (0.4) \| | 6630 distinct values | 0  (0.0%) |
| 2 | inequality  [numeric] | \| Mean (sd) : 0.2 (1.3) \| \| --- \| \| min ≤ med ≤ max: \| \| -3.2 ≤ 0 ≤ 5.2 \| \| IQR (CV) : 1.7 (6.5) \| | 6681 distinct values | 0  (0.0%) |
| 3 | expansion  [numeric] | \| Mean (sd) : 3.1 (1.9) \| \| --- \| \| min ≤ med ≤ max: \| \| -3.4 ≤ 3.1 ≤ 10.9 \| \| IQR (CV) : 2.6 (0.6) \| | 6678 distinct values | 0  (0.0%) |
| 4 | dependency  [numeric] | \| Mean (sd) : 0.4 (0.1) \| \| --- \| \| min ≤ med ≤ max: \| \| -0.1 ≤ 0.4 ≤ 1 \| \| IQR (CV) : 0.2 (0.3) \| | 6674 distinct values | 0  (0.0%) |
| 5 | cohort  [factor] | \| 1. 1940 \| \| --- \| \| 2. 1950 \| \| 3. 1960 \| \| 4. 1970 \| \| 5. 1980 \| | \| 1189 \| ( \| 17.7% \| ) \| \| --- \| --- \| --- \| --- \| \| 1259 \| ( \| 18.7% \| ) \| \| 1275 \| ( \| 19.0% \| ) \| \| 1271 \| ( \| 18.9% \| ) \| \| 1731 \| ( \| 25.7% \| ) \| | 0  (0.0%) |
| 6 | fragile  [factor] | \| 1. No \| \| --- \| \| 2. Yes \| | \| 5890 \| ( \| 87.6% \| ) \| \| --- \| --- \| --- \| --- \| \| 835 \| ( \| 12.4% \| ) \| | 0  (0.0%) |
| 7 | developing  [factor] | \| 1. No \| \| --- \| \| 2. Yes \| | \| 2227 \| ( \| 33.1% \| ) \| \| --- \| --- \| --- \| --- \| \| 4498 \| ( \| 66.9% \| ) \| | 0  (0.0%) |
| 8 | region  [factor] | \| 1. East Asia & Pacific \| \| --- \| \| 2. Europe & Central Asia \| \| 3. Latin America & Caribbean \| \| 4. Middle East & North Afric \| \| 5. North America \| \| 6. South Asia \| \| 7. Sub-Saharan Africa \| | \| 837 \| ( \| 12.4% \| ) \| \| --- \| --- \| --- \| --- \| \| 2788 \| ( \| 41.5% \| ) \| \| 687 \| ( \| 10.2% \| ) \| \| 468 \| ( \| 7.0% \| ) \| \| 120 \| ( \| 1.8% \| ) \| \| 332 \| ( \| 4.9% \| ) \| \| 1493 \| ( \| 22.2% \| ) \| | 0  (0.0%) |
| 9 | mom  [factor] | \| 1. No \| \| --- \| \| 2. Yes \| | \| 5091 \| ( \| 75.7% \| ) \| \| --- \| --- \| --- \| --- \| \| 1634 \| ( \| 24.3% \| ) \| | 0  (0.0%) |
| 10 | daughter  [factor] | \| 1. No \| \| --- \| \| 2. Yes \| | \| 4483 \| ( \| 66.7% \| ) \| \| --- \| --- \| --- \| --- \| \| 2242 \| ( \| 33.3% \| ) \| | 0  (0.0%) |

*Notes: The sample size N = 6725. The dataset is adopted from the Global Database on Intergenerational Mobility (GDIM) 2023. In it, mobility = CAT, inequality = SDc – SDp, expansion = MEANc – MEANp, family = COR, cohort = cohort, fragile = fragile, developing = incgroup2 (developing economies), culture = region_noHICgroup, mom = parent (mom), daughter = child (daughter).*
