# Supplementary material for: What really matters for global intergenerational mobility?
Source: PLoS One. 2024 Jun 20;19(6):e0302173. doi: 10.1371/journal.pone.0302173 (PMC11189229; doi:10.1371/journal.pone.0302173)
Supplement: S3 Appendix — (DOCX) [file pone.0302173.s003.docx]

**Appendix 3. The modelled dataset**

| **No** | **Variable** | **Stats / Values** | **Freqs (% of Valid)** | **Missing** |
| --- | --- | --- | --- | --- |
| 1 | mobility  [numeric] | \| Mean (sd) : 1.5 (0) \| \| --- \| \| min ≤ med ≤ max: \| \| 1.4 ≤ 1.5 ≤ 1.6 \| \| IQR (CV) : 0 (0) \| | 6633 distinct values | 0  (0.0%) |
| 2 | inequality  [numeric] | \| Mean (sd) : 1.5 (0.3) \| \| --- \| \| min ≤ med ≤ max: \| \| 0.2 ≤ 1.5 ≤ 2.3 \| \| IQR (CV) : 0.4 (0.2) \| | 6681 distinct values | 0  (0.0%) |
| 3 | expansion  [numeric] | \| Mean (sd) : 2 (0.3) \| \| --- \| \| min ≤ med ≤ max: \| \| 0 ≤ 2 ≤ 2.7 \| \| IQR (CV) : 0.3 (0.1) \| | 6678 distinct values | 0  (0.0%) |
| 4 | dependency  [numeric] | \| Mean (sd) : 1.6 (0) \| \| --- \| \| min ≤ med ≤ max: \| \| 1.5 ≤ 1.6 ≤ 1.7 \| \| IQR (CV) : 0 (0) \| | 6674 distinct values | 0  (0.0%) |
| 5 | cohort1950  [numeric] | \| Min : 0 \| \| --- \| \| Mean : 0.2 \| \| Max : 1 \| | \| 0 \| : \| 5466 \| ( \| 81.3% \| ) \| \| --- \| --- \| --- \| --- \| --- \| --- \| \| 1 \| : \| 1259 \| ( \| 18.7% \| ) \| | 0  (0.0%) |
| 6 | cohort1960  [numeric] | \| Min : 0 \| \| --- \| \| Mean : 0.2 \| \| Max : 1 \| | \| 0 \| : \| 5450 \| ( \| 81.0% \| ) \| \| --- \| --- \| --- \| --- \| --- \| --- \| \| 1 \| : \| 1275 \| ( \| 19.0% \| ) \| | 0  (0.0%) |
| 7 | cohort1970  [numeric] | \| Min : 0 \| \| --- \| \| Mean : 0.2 \| \| Max : 1 \| | \| 0 \| : \| 5454 \| ( \| 81.1% \| ) \| \| --- \| --- \| --- \| --- \| --- \| --- \| \| 1 \| : \| 1271 \| ( \| 18.9% \| ) \| | 0  (0.0%) |
| 8 | cohort1980  [numeric] | \| Min : 0 \| \| --- \| \| Mean : 0.3 \| \| Max : 1 \| | \| 0 \| : \| 4994 \| ( \| 74.3% \| ) \| \| --- \| --- \| --- \| --- \| --- \| --- \| \| 1 \| : \| 1731 \| ( \| 25.7% \| ) \| | 0  (0.0%) |
| 9 | fragileYes  [numeric] | \| Min : 0 \| \| --- \| \| Mean : 0.1 \| \| Max : 1 \| | \| 0 \| : \| 5890 \| ( \| 87.6% \| ) \| \| --- \| --- \| --- \| --- \| --- \| --- \| \| 1 \| : \| 835 \| ( \| 12.4% \| ) \| | 0  (0.0%) |
| 10 | developingYes  [numeric] | \| Min : 0 \| \| --- \| \| Mean : 0.7 \| \| Max : 1 \| | \| 0 \| : \| 2227 \| ( \| 33.1% \| ) \| \| --- \| --- \| --- \| --- \| --- \| --- \| \| 1 \| : \| 4498 \| ( \| 66.9% \| ) \| | 0  (0.0%) |
| 11 | regionEuropeCentralAsia  [numeric] | \| Min : 0 \| \| --- \| \| Mean : 0.4 \| \| Max : 1 \| | \| 0 \| : \| 3937 \| ( \| 58.5% \| ) \| \| --- \| --- \| --- \| --- \| --- \| --- \| \| 1 \| : \| 2788 \| ( \| 41.5% \| ) \| | 0  (0.0%) |
| 12 | regionLatinAmericaCaribbean  [numeric] | \| Min : 0 \| \| --- \| \| Mean : 0.1 \| \| Max : 1 \| | \| 0 \| : \| 6038 \| ( \| 89.8% \| ) \| \| --- \| --- \| --- \| --- \| --- \| --- \| \| 1 \| : \| 687 \| ( \| 10.2% \| ) \| | 0  (0.0%) |
| 13 | regionMiddleEastNorthAfrica  [numeric] | \| Min : 0 \| \| --- \| \| Mean : 0.1 \| \| Max : 1 \| | \| 0 \| : \| 6257 \| ( \| 93.0% \| ) \| \| --- \| --- \| --- \| --- \| --- \| --- \| \| 1 \| : \| 468 \| ( \| 7.0% \| ) \| | 0  (0.0%) |
| 14 | regionNorthAmerica  [numeric] | \| Min : 0 \| \| --- \| \| Mean : 0 \| \| Max : 1 \| | \| 0 \| : \| 6605 \| ( \| 98.2% \| ) \| \| --- \| --- \| --- \| --- \| --- \| --- \| \| 1 \| : \| 120 \| ( \| 1.8% \| ) \| | 0  (0.0%) |
| 15 | regionSouthAsia  [numeric] | \| Min : 0 \| \| --- \| \| Mean : 0 \| \| Max : 1 \| | \| 0 \| : \| 6393 \| ( \| 95.1% \| ) \| \| --- \| --- \| --- \| --- \| --- \| --- \| \| 1 \| : \| 332 \| ( \| 4.9% \| ) \| | 0  (0.0%) |
| 16 | regionSubSaharanAfrica  [numeric] | \| Min : 0 \| \| --- \| \| Mean : 0.2 \| \| Max : 1 \| | \| 0 \| : \| 5232 \| ( \| 77.8% \| ) \| \| --- \| --- \| --- \| --- \| --- \| --- \| \| 1 \| : \| 1493 \| ( \| 22.2% \| ) \| | 0  (0.0%) |
| 17 | momYes  [numeric] | \| Min : 0 \| \| --- \| \| Mean : 0.2 \| \| Max : 1 \| | \| 0 \| : \| 5091 \| ( \| 75.7% \| ) \| \| --- \| --- \| --- \| --- \| --- \| --- \| \| 1 \| : \| 1634 \| ( \| 24.3% \| ) \| | 0  (0.0%) |
| 18 | daughterYes  [numeric] | \| Min : 0 \| \| --- \| \| Mean : 0.3 \| \| Max : 1 \| | \| 0 \| : \| 4483 \| ( \| 66.7% \| ) \| \| --- \| --- \| --- \| --- \| --- \| --- \| \| 1 \| : \| 2242 \| ( \| 33.3% \| ) \| | 0  (0.0%) |

*Notes: The sample size N = 6725. This dataset has been transformed from the pre-modelled dataset. All numeric variables have been logged with an offset or a constant (which is calculated as the absolute value of the minimum value in the whole dataset plus 1). All categorical variables have been dummy-encoded. Previously, mobility was country-centred, meaning that the country mean was subtracted from each observation within that country. This technique effectively removes fixed effects.*
