# Supplementary material for: What really matters for global intergenerational mobility?
Source: PLoS One. 2024 Jun 20;19(6):e0302173. doi: 10.1371/journal.pone.0302173 (PMC11189229; doi:10.1371/journal.pone.0302173)
Supplement: S4 Appendix — (DOCX) [file pone.0302173.s004.docx]

**Appendix 4. Does education expansion work for promoting upward mobility?**

| model | var | estimate | std_error | t_value | p_value |
| --- | --- | --- | --- | --- | --- |
| Partialling-out Lasso | inequality | -0.011 | 0.001 | -10.827 | 0.000 |
| Partialling-out Lasso | expansion | 0.060 | 0.001 | 41.453 | 0.000 |
| Partialling-out Lasso | dependency | -0.126 | 0.011 | -11.409 | 0.000 |
| Double ML | inequality | -0.009 | 0.001 | -7.880 | 0.000 |
| Double ML | expansion | 0.054 | 0.001 | 39.038 | 0.000 |
| Double ML | dependency | -0.100 | 0.011 | -8.750 | 0.000 |

*Notes: The sample size N = 6725. This table is referring to Figure 4*
