# Supplementary material for: What really matters for global intergenerational mobility?
Source: PLoS One. 2024 Jun 20;19(6):e0302173. doi: 10.1371/journal.pone.0302173 (PMC11189229; doi:10.1371/journal.pone.0302173)
Supplement: S5 Appendix — (DOCX) [file pone.0302173.s005.docx]

**Appendix 5. Does gender bias exist in terms of upward mobility?**

| model | var | estimate | std_error | t_value | p_value |
| --- | --- | --- | --- | --- | --- |
| Partialling-out Lasso | daughterYes | 0.053 | 0.027 | 1.931 | 0.054 |
| Partialling-out Lasso | daughterYes:inequality | -0.015 | 0.002 | -7.885 | 0.000 |
| Partialling-out Lasso | daughterYes:expansion | -0.001 | 0.002 | -0.442 | 0.658 |
| Partialling-out Lasso | daughterYes:dependency | -0.027 | 0.017 | -1.555 | 0.120 |
| Partialling-out Lasso | daughterYes:cohort1950 | 0.003 | 0.001 | 1.717 | 0.086 |
| Partialling-out Lasso | daughterYes:cohort1960 | 0.007 | 0.001 | 4.806 | 0.000 |
| Partialling-out Lasso | daughterYes:cohort1970 | 0.011 | 0.001 | 7.124 | 0.000 |
| Partialling-out Lasso | daughterYes:cohort1980 | 0.019 | 0.001 | 13.673 | 0.000 |
| Partialling-out Lasso | daughterYes:fragileYes | -0.003 | 0.001 | -1.859 | 0.063 |
| Partialling-out Lasso | daughterYes:developingYes | -0.003 | 0.001 | -2.229 | 0.026 |
| Partialling-out Lasso | daughterYes:regionEuropeCentralAsia | 0.005 | 0.001 | 3.341 | 0.001 |
| Partialling-out Lasso | daughterYes:regionLatinAmericaCaribbean | 0.003 | 0.002 | 1.467 | 0.142 |
| Partialling-out Lasso | daughterYes:regionMiddleEastNorthAfrica | -0.005 | 0.002 | -2.377 | 0.017 |
| Partialling-out Lasso | daughterYes:regionNorthAmerica | 0.016 | 0.004 | 4.409 | 0.000 |
| Partialling-out Lasso | daughterYes:regionSouthAsia | -0.002 | 0.002 | -0.973 | 0.331 |
| Partialling-out Lasso | daughterYes:regionSubSaharanAfrica | -0.005 | 0.002 | -3.254 | 0.001 |
| Partialling-out Lasso | daughterYes:momYes | 0.001 | 0.001 | 0.624 | 0.533 |
| Double ML | daughterYes | -0.005 | 0.001 | -10.165 | 0.000 |

*Notes: The sample size N = 6725. This table is referring to Figure 7*
