# Supplementary material for: What really matters for global intergenerational mobility?
Source: PLoS One. 2024 Jun 20;19(6):e0302173. doi: 10.1371/journal.pone.0302173 (PMC11189229; doi:10.1371/journal.pone.0302173)
Supplement: Attachment — Submitted filename: Response to reviewers.docx [file pone.0302173.s006.docx]

Please use the space provided to explain your answers to the questions above. You may also include additional comments for the author, including concerns about dual publication, research ethics, or publication ethics. (Please upload your review as an attachment if it exceeds 20,000 characters)

**Reviewer #1:** The authors have analyzed the relationship between education and gender inequality and intergenerational mobility using data from the Global Database on Intergenerational Mobility for 153 countries and cohorts born between the 1940s and 1980s. The paper specifically looks at two questions: first, whether education expansion policies mitigate the negative impacts of education inequality and parental influence on upward mobility; and second, whether gender inequality affect upward intergenerational mobility.

The authors have taken a painstakingly long exercise to answer questions which seem to have been thoroughly studied in literature and have been concretely established by multiple empirical studies. In other words, the research gaps this paper tries to fill are not clear. The authors also do not clarify how an analysis using the cohorts from 1940 to 1980 provides significant learnings for today’s policy and practice.

Some specific comments are below:

• The introduction is broken in two parts to justify the two research questions. Authors touch upon Great Gatsby Curve, importance of education, welfare-regime of the state, among the other predictors of inter-generational mobility, but the research question framed is limited only to education expansion policies given the inequality and parental dependency – even when authors cite other literature which investigate these relations. The literature cited do not justify the question framed.

*🡪 I have revised the introduction. Question 1 is based on the research by Deirdre Bloome et al., published in the American Sociological Review, which discusses how education expansion policies may have their effectiveness reduced due to social inequality and parental dependency. Furthermore, the effectiveness of education expansion is also questioned by Zhong (published in the Economica journal) with the phenomenon of 'over-education'. In the complex relationship between these three factors and social mobility, I pose the question of whether the true effect of education expansion is positive.*

• The second part of the introduction is supposedly about gender inequality. But line 68 – 76 mention other studies that study inequality.

*🡪 Initially, I intended to write broadly about social inequality, subsequently highlighting gender inequality as the most concerning factor. However, considering the introduction was becoming excessively lengthy, I decided to truncate it.*

• In line 91: Authors mention that there is a research gap regarding understanding gender inequalities that emanate from cultural and social norms. This is an incorrect assumption as there have been plethora of research now on understanding the multi-dimensionality of gender as well as its fluidity. Authors go on to cite evidence of multidimensional impact of gender but frame the research question without any mention of the same.

*🡪 I have clarified Question 2. Despite numerous studies indicating the severe existence of gender inequality in intergenerational mobility, renowned economist Alberto Alesina et al. [31] conducted a meta-data analysis in Africa and found no evidence of systematic gender gaps in intergenerational mobility. Furthermore, a World Bank report (Fair Progress? Economic Mobility across Generations around the World) has indicated that gender inequality has been significantly reduced across generations. Therefore, this study aims to reassess the importance of gender effects from a multidimensional perspective (space, time, national and regional context). To compare the importance between factors, the effect sizes have been standardized, as described in the materials and methods section.*

• Data and methods:

o The authors do not justify why they use education as an indicator for mobility and not income or wealth like many of the literature they cite.

*🡪 Upward mobility, the main concept of this study, refers to the degree of absolute mobility in the GDIM. This indicator shows the probability that a child achieves a higher level of education than their parent. Education is a suitable proxy for upward mobility because people value equal opportunities (e.g., access to education) more than equal outcomes (e.g., income or wealth) [34]. Additionally, income is a problematic variable to compare across countries and generations due to different standards of living and inflation. Education, by contrast, is a more stable and comparable measure, and a crucial predictor of income [35].*

o The indicator construction overall is unclear in the article.

*🡪 I have rewritten this section, where each variable in the model is referred to with specific theories and studies, and these variables are based on the definitions from the Global Database on Intergenerational Mobility. Additionally, to clarify the construction of the variables, I have developed an analytical framework based on past literature, please refer to Figure 1.*

o Education expansion does not necessarily reflect education policy, but authors use them interchangeably without justification.

*🡪 Thanks, I have edited the wordings.*

o The last cohort of the Global Database on Intergenerational Mobility is 1980 whereas, authors include indicators of region and ‘fragility’ from a 2020 database. Other than the simple confirmation that the indicators of interest vary significantly by these country types, there is no concrete justification as to how a 2020 classification of countries apply to a 1980 cohort when there has been significant overhaul of policies both internally and globally.

🡪 *Please note that the term 'Fragile' is applied to the most recent survey period for the GDIM 2023 dataset, which surveys individuals born between the 1940s and 1980s, rather than data applied during the years 1940-1980s. The 'Fragile' variable is used in the GDIM 2023 and is specifically described in the following study:*

*van der Weide, R., Lakner, C., Mahler, D. G., Narayan, A., & Gupta, R. (2024). Intergenerational mobility around the world: A new database. Journal of Development Economics, 166, 103167.*

o Authors talk about intergenerational mobility in terms of education without much justification. How does the probability of a child surpassing their parent's educational category, given that the parent does not have tertiary education reflect “expansion policies”? It can also reflect changes in country’s overall economic structure – and therefore, marginal propensity to consume.

🡪 *In the education level classification of the GDIM, tertiary education is considered the highest level. This implies that even if children attain tertiary education, it cannot necessarily be viewed as upward mobility. Furthermore, "the expansion of education can potentially elevate the educational levels of a nation as a whole, but it does not necessarily imply a transformation in the internal social structure of that nation."*

*Upward mobility, the main concept of this study, refers to the degree of absolute mobility in the GDIM. This indicator shows the probability that a child achieves a higher level of education than their parent. Education is a suitable proxy for upward mobility because people value equal opportunities (e.g., access to education) more than equal outcomes (e.g., income or wealth) [34]. Additionally, income is a problematic variable to compare across countries and generations due to different standards of living and inflation. Education, by contrast, is a more stable and comparable measure, and a crucial predictor of income [35].*

*Education expansion, as referenced in the study of Deirdre Bloome et al. [13], represents the difference between the mean years of schooling of children and the mean years of schooling of parents. It signifies the magnitude of educational expansion within a specific population. Higher values of education expansion indicate a greater increase in educational attainment among younger generations, potentially contributing to upward mobility. It should be noted that the expansion of education can potentially elevate the educational levels of a nation as a whole, but it does not necessarily imply a transformation in the internal social structure of that nation.*

o For both q1 and q2 models, there is no clarity on what the control variables are other than the indicators of interest. For any model on education, a number of predictors from both supply and demand side should be controlled for. Authors need to justify if no other controls are added.

*🡪 In each figure, I have included notes below. "The sample size N = 6,725. In this study, the outcome variable is upward mobility. For each estimate, the treatment variables (target variables) are either (education) inequality, (education) expansion, or (parental) dependency. The confounding variables consist of all other variables present in the dataset, which are not directly considered treatment or outcome variables." This implies that the number of control variables is large, and causal machine learning only utilizes these variables for the learning process and does not generate causal inferences for these variables. Therefore, its results only show the impact of the target variables.*

o Authors note the contradictory results (Line 387-389: maternal education is strongly associated with immobility; but children are less dependent on their mother's education) but do not explain, interpret or discuss these findings at all.

*🡪 Thanks, I have rerun the model and reinterpreted the research results.*

o Authors make large assumptions based on country/region effects without clarifying or justifying these assumptions. If region specific results vary from overall results, are these results robust?

*🡪 Please refer to Figures 7 & 8. These variables are considered as contextual factors and are analyzed in the World Bank report (Fair Progress? Economic Mobility across Generations around the World). Additionally, to check robustness, I have performed an ANOVA test (see Figure 2) and also interpreted these results in the model outcomes in Figure 7, and cross-referenced the actual data in some countries in Figure 8. The results of the causal machine learning are robust with cross-fitting (see the materials and methods section).*

• The results dominate the write-up and discussion section does not go into interpretation of the results or try to place hypotheses from the counter-intuitive results. The methodology section also does not justify why the machine learning techniques were required or why a violin plot was needed to describe the data.

*🡪 Please refer to “Figure 3. How do causal machine-learning techniques address confounding problems?” Machine learning approaches aim to measure true effects when there exists a complex web of relationships among explanatory variables (as presented in the analytical framework). The violin plot is used to visualize the results of the ANOVA analysis as mentioned in the manuscript. Furthermore, I have rewritten the results and discussion sections to clarify the research findings.*

• Overall, the paper is poorly structured with no clear distinction between methodology, results, discussion, and conclusion. The results are also not placed in the larger context to clarify what this article is adding to a very old discussion.

*🡪 Please refer to the following section in the manuscript. The contribution of this research lies in "reassessing the roles of parental dependency and gender bias in intergenerational mobility, especially when parental dependency is currently underestimated, and gender bias is overemphasized."*

*This study aims to measure the significance of factors such as education expansion, social inequality, parental dependency, and gender effects on intergenerational mobility. As depicted in Figure 1, social inequality and parental dependency have long been recognized as major barriers to upward mobility (denoted as 1 and 2), and they also undermine efforts in education expansion to promote upward mobility (denoted as 3). Furthermore, the question regarding the effectiveness of education expansion itself was raised by Hai Zhong [14] in the context where over-education might not facilitate an individual's upward mobility. While education expansion, social inequality, and parental dependency have a direct impact on upward mobility, gender effects fundamentally influence upward mobility through indirect mechanisms in the family, society, and context. As gender differences are gradually eliminated across generations, and as Alberto Alesina et al. [31] no longer found strong evidence of gender inequality in Africa in recent research, these findings necessitate a revisit of the extent of gender inequality in intergenerational mobility, especially when its multidimensionality and dynamics challenge traditional methods. Utilizing causal machine learning recently developed by Chernozhukov et al. [32] to address confounding problems, I re-examine the true effect of education expansion, social inequality, parental dependency, and gender effects in complex multidimensional relationships. The originality of this research lies in demonstrating that promoting social mobility primarily involves lowering the level of parental dependency, rather than addressing social inequalities (such as education inequality or gender bias) - as many studies are concerned with - or education expansion - as governments are implementing*

Other than these, authors need to use consistent language, not use "females" when analyzing gender but use "women" and follow the structure of a scientific article.

*🡪 Thanks, I edited the wordings.*

The manuscript needs to be thoroughly read and restructured before it can be published.

**Reviewer #2:** This study used data from the Global Database on Intergenerational Mobility for 153 countries and those born between the 1940s and 1980s to examine the relationship between education and gender inequality on one hand, and intergenerational mobility on the other.

The aim was to:

1. Assess the effectiveness of education policies in promoting upward mobility

2. Investigate the direct and indirect effects of gender inequality on mobility outcomes.

The study found intricate interconnections among significant factors, such as educational expansion, educational inequality, and parental dependency, as they collectively shape mobility outcomes.

Even though what the study found is not so novel and we already know these, the author need to take time to reorganize the sections to make them more clearer and be more targeted in the recommendations. I have therefore suggested some actions below in this direction.

Analysis

1. With regards to your analysis, could you pull out the data on one or two countries in Sub-Saharan Africa and show how they contradict the East Asian and European countries? It will be good to se that analysis as shown for India, China, Denmark and the others.

*🡪 Thanks, I have added Figure 8 to demonstrate this.*

Recommendations

2. Please unpick your recommendations and show them distinctively. For example:

- At which level of education would this investment you call for be made? Would grater educational investment be required at the basic, primary, pre-tertiary or at the tertiary levels? And please note, these must bring maximum impact.

*🡪 I have completely rewritten the research and discussion sections. As the above question is not within the scope of my research, I am unable to provide an accurate answer. My research solely aims to measure the importance of factors such as education expansion, education inequality, parental expansion, and gender effects in relation to intergenerational mobility.*

3. The greater obstacles women face in achieving upward mobility should be addressed with which measures?

*🡪 My research has clearly indicated that gender inequality has been significantly reduced across generations (though it still remains at a relatively small level). This is entirely consistent with a World Bank report (Fair Progress? Economic Mobility across Generations around the World). The gender effect is multidimensional and influenced by social norms and cultures. My study suggests that, to promote social mobility, the most crucial change is to alter the tradition of "like father, like son".*

4. Is it only high educational levels that promote upward mobility? How about income levels of parents? And places such as India, how about extended family support systems that tend to enable people’s social mobility.

*🡪 As clearly stated in the methods section, this study measures upward mobility through education levels (not income), and educational expansion can enhance intergenerational mobility to a certain extent. However, its effectiveness is partially offset by educational inequality and may be ineffective if high levels of parental influence persist. Furthermore, my research is generally applicable at the global level (by excluding country-specific effects), making it impossible to make specific assertions for a particular country like India, as social mobility is heavily dependent on the specific characteristics of social norms and cultures, as analyzed in the article.*
